# Food Restriction Induces Changes in Ovarian Folliculogenesis, Cell Proliferation, Apoptosis, and Production of Regulatory Peptides in Rabbits

**DOI:** 10.3390/ani15091282

**Published:** 2025-04-30

**Authors:** Imane Hadjadj, Zuzana Fabova, María-Luz García, Iván Agea, Barbora Loncová, Martin Morovic, Peter Makovicky, María-José Argente, Alexander V. Sirotkin

**Affiliations:** 1 Instituto de Investigación e Innovación Agroalimentario y Agroambiental (CIAGRO), Universidad Miguel Hernández de Elche, Ctra de Beniel Km 3.2, 03312 Orihuela, Spain; imane.hadjadj@alu.umh.es (I.H.); iagea@umh.es (I.A.); mj.argente@umh.es (M.-J.A.); 2 Department of Zoology and Anthropology, Constantine the Philosopher University, Tr. A 14 Hlinku 1, 949 74 Nitra, Slovakiammorovic@ukf.sk (M.M.); asirotkin@ukf.sk (A.V.S.); 3 Department of Histology and Embryology, Faculty of Medicine, University of Ostrava, Dvořákova 138/7, 701 03 Ostrava, Czech Republic; pmakovicky@email.cz; 4 Research Institute, Biomedical Research Centre of the Slovak Academy of Sciences, Dúbravská cesta 9, 814 39 Bratislava, Slovakia

**Keywords:** apoptosis, food restriction, hormone, ovarian follicle, rabbit

## Abstract

The aim of this study is to examine the influence of food restriction on rabbit ovarian functions. A total of eight females were fed ad libitum (NF), while eight females were subjected to 50% food restriction (RF). One month later, all females were euthanized. Weights and lengths of ovaries and uterine horns were measured. Representative parts of the ovaries were subjected to histomorphometry analysis of folliculogenesis. Granulosa cells were isolated and cell viability, proliferation (accumulation of PCNA, cyclin B1, and BrdU-positive cells), apoptosis (accumulation of bax, caspase 3, and DNA fragmentation) were evaluated. Granulosa cells were subjected to proteomic analysis by using the nano HPLC-Chip-MS/MS method. Estradiol and progesterone release by ovarian and granulosa cells was assessed by ELISA. Ovarian and uterine horn weights were lower in RF than NF. The diameter of follicles and oocytes and the thickness of the theca and granulosa cells were higher in RF than NF. RF showed a lower percentage of cells containing bax and caspase 3, occurrence of DNA fragmented cells, and estradiol and progesterone. RF had higher incorporation of BrdU, a higher proportion of cells containing PCNA and cyclin B1, and a lower percentage of viable cells. RF produced more specific proteins than NF, including peptides involved in cell differentiation, proliferation/division, mitotic cell cycle, and GTP-ase activity. In conclusion, food restriction can activate reproduction by (1) selection of the growing primordial follicles, (2) better transformation of secondary to preovulatory follicles, (3) increasing growth of oocytes, (4) increasing proliferation and decreasing apoptosis in granulosa cells, (5) changes in ovarian secretory activity, and (6) changes in the number of peptides.

## Simple Summary

In prolific species such as rabbits, knowing how to improve their productive parameters through ovarian changes is of special importance. In this study, a 50% food restriction is proposed to induce changes in folliculogenesis, cell proliferation, and apoptosis. Females fed with 50% caloric restriction can improve fecundity by selection of the growing primordial ovarian follicles, better transformation of secondary to preovulatory follicles, and increased growth (cytoplasmic maturation) of oocytes. Caloric restriction can activate rabbit female reproduction by increasing proliferation and decreasing apoptosis in ovarian granulosa cells, changes in ovarian secretory activity, and changes in the number of peptides involved in cell differentiation, proliferation/division (cytoskeletal proteins), and adhesion.

## 1. Introduction

Nutrition is one of the main factors influencing reproductive efficiency, especially in female mammals. Several studies have demonstrated that variations in energy feed intake may have a direct or indirect effect on female reproduction of farm animals through alterations in GnRH secretion, delay or inhibition of LH peak and estradiol-17β, and progesterone concentration variations (in heifers, [1]; in gilts, [2]; in ewes, [3]; in does, [4]). Likewise, they have been shown to affect ovarian follicle development and follicular characteristics in heifers [5] and ewes [6].

In commercial rabbit production, different feeding programs, such as ad libitum feeding or feed restriction, are common practices [7] to optimize the production system. However, the available data concerning consequences and mechanisms of influence of feed restriction on rabbit ovarian functions, folliculogenesis, and, ultimately, fertility are unclear and contradictory. Some previous studies have demonstrated that food restriction does not affect the number of developing ovarian follicles, ovulation rate, oocyte quality, and kindling rate in rabbits [8,9,10]. Other studies have shown that caloric feed restriction before mating, applied intermittently or continuously, promotes subsequent oocyte maturation, fertility, and kindling rate [11,12,13,14]. These food-restriction-induced changes in rabbit fecundity could be mediated by changes in the state and release of hormones by ovarian cells. At least, Sirotkin et al. [14] and Harrath et al. [15] showed that food restriction can modulate plasma levels of IGF-I, leptin, and progesterone (but not estradiol) and proliferation (accumulation of PCNA and cyclin B1) and reduction of apoptosis (accumulation of bax) in rabbit ovarian granulosa cells. Nevertheless, it remains unclear whether food restriction can affect other ovarian cell functions such as viability, production of steroid hormones, and the resulting growth of ovarian follicles and oocytes. Moreover, from a practical point of view, understanding how dietary restriction affects the mechanisms that regulate fertility can be of relevance for optimizing production by reducing feed costs.

The primary objective of this study is to examine the role of nutritional status in rabbit fecundity. Specifically, we aim to compare the state and function of ovarian cells and follicles between rabbits subjected to food restriction and those fed ad libitum. The following parameters are examined: the weights and lengths of ovaries and the uterus, histomorphological assessment of ovarian follicles and their components, viability, proliferation, apoptosis, and the synthesis of steroid hormones and regulatory peptides essential for regulating basic functions of ovarian cells.

## 2. Materials and Methods

### 2.1. Biological Material

The females belonged to generation 17 of a synthetic maternal line genetically selected to homogenize litter size at birth. Details of the breed are indicated in [16]. The animals were bred and housed in individual cages at the farm of Miguel Hernández University (Orihuela, Spain). A total of 16 multiparous nonlactating female rabbits were kept under a constant photoperiod of 16:8 h, controlled ventilation, temperature (23–25 °C), and relative humidity (65–80%). All the animals were fed one time per day with a standard feed (16.3% crude protein, 15% crude fiber, 3.2% ether extract, 8.9% ash, 0.56% phosphor, and 0.23% sodium; CUNILACTAL, NANTA S.A., Las Palas, Murcia, Spain). Does were mated for the first time at 18 weeks of age and, later, 10 days after delivery. The nonreceptive does were mated again the following week. Pregnancy diagnosis was made by abdominal palpation 12 days after natural mating, and the kits were weaned at 28 days of age.

Before the experiments, females were randomly divided into two groups (eight animals per group) at the end of the 5th lactation: (1) nonrestricted females (received full food dose, NF) and (2) subjected to 50% food restriction (received 50% of food dose, RF). The experiment lasted one month. Daily feed intake was 308 g/day for the NF group and 151 g/day for the RF group. Weights at the beginning and end of the experiment for both groups were recorded. All females were nonlactating and at the same estrus stage at the end of the experiment. Then, they were euthanized by intravenous administration of sodium thiopental in a dose of 50 mg/kg of body weight (Thiobarbital, B. Braun Medical S.A., Barcelona, Spain). The entire reproductive tract was immediately removed, and the weight and length of the right and left ovaries and uterine horns were measured. Then, a quarter of the ovaries were submerged in 4% paraformaldehyde, and the remainder of the ovaries were submerged in PBS for subsequent analysis.

### 2.2. Histological Processing and Analysis of Ovaries

**Figure 1:**
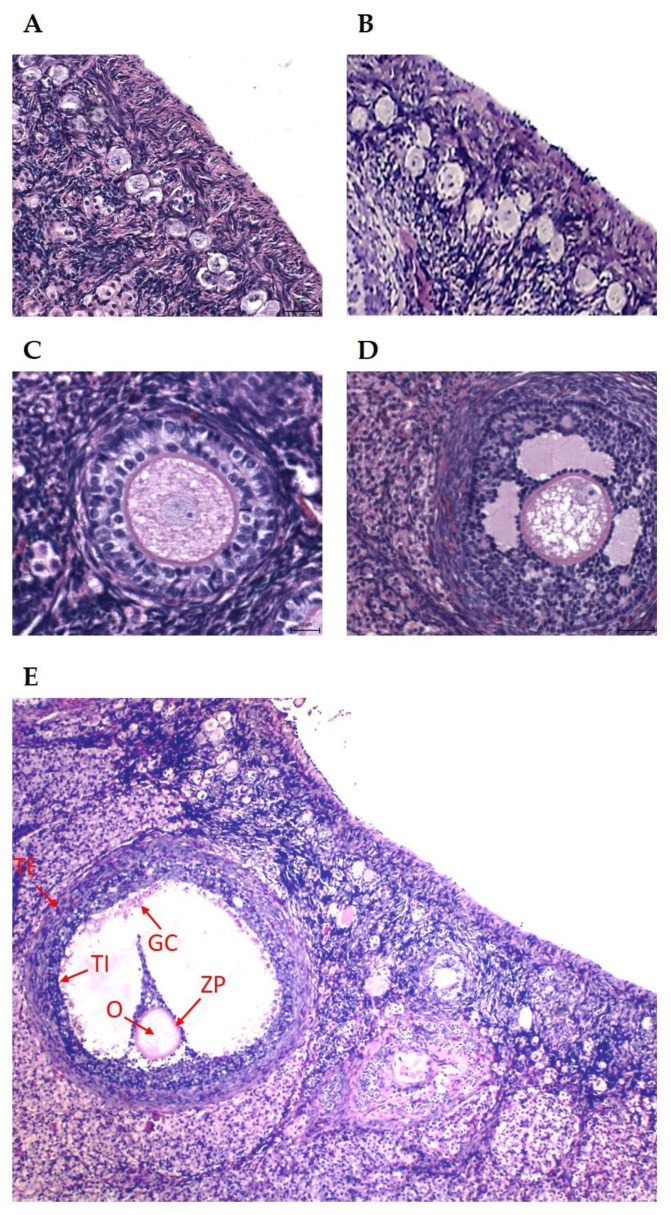
(**A**) Dormant primordial follicles, HE: 400×; (**B**) growing primordial follicles, HE: 400×; (**C**) primary (preantral) follicle, HE: 400×; (**D**) secondary (antral) follicle, HE: 200×; (**E**) preovulatory follicle. The arrows indicate the TE (theca externa), TI (theca interna), GC (granulosa cells), ZP (zona pellucida), and O (oocyte). HE: 100×.

The ovarian samples were fixed immediately after animal slaughter in 10% formalin solution (Sigma-Aldrich Corp., St. Louis, MO, USA) for a minimum of 24 h, with longer fixation producing formalin artifacts. From each animal, one ovary was sent to a histopathological laboratory, and from each ovary, several sections were made to obtain serial slices. Only follicles with a clearly visible nucleus were included in the study. Afterward, they were processed by standard histological methods using an automated tissue processor (Leica ASP6025, Leica Microsystems, Wetzlar, Germany) and were then embedded in paraffin blocks using a Leica EG 1150H paraffin embedding station (Leica Microsystems, Wetzlar, Germany). A microtome (Leica RM2255, Leica Microsystems, Wetzlar, Germany) was used to cut sections in thickness of three to five μm from each sample, and every fifth slice was mounted on standard glass slides. The sections were stained with hematoxylin–eosin (Bamed s.r.o., Litvínovice, Czech Republic). The identification, classification, and counting of primary and secondary ovarian follicles, as well as large nonovulated hemorrhagic follicles, were performed as described previously [17,18]. The number of follicles examined in each section varied from one to fifty per section. The follicles were classified according to their morphological state (presence of antrum, presence and number of layers of granulosa cells), described in the general *European Histology* book [19]. The number of granulosa cell layers was used as the criterion to distinguish primary from secondary ovarian follicles (Figure 1). All the ovarian follicles, irrespective of size and signs of atresia, were analyzed. The ovarian morphology was described, and the average thickness of primary, secondary, and hemorrhagic anovulatory follicles was measured. Follicles with morphological signs of degeneration (blebbing, deformation of follicle, follicular cells and their nuclei, appearance of apoptotic bodies, pyknosis, and vacuolization of cytoplasm) were considered atretic/degenerated. An Axio Scan.Z1 slide scanner (Zeiss, Oberkochen, Germany) was used for measurements, and histological evaluation was conducted using a Carl Zeiss Axio Scope A1 microscope (Zeiss). Only follicles with clearly visible oocytes were measured, and all follicles were measured twice.

### 2.3. Preparation, Culture, and Processing of Ovarian Granulosa Cells

In the initial series of experiments designed to evaluate the viability, proliferation, apoptosis, and secretory activity of ovarian cells, the following methodology was employed. Ovaries were placed into Petri dishes containing a culture medium. Ovarian follicles, visibly ranging from 0.5 to 2.0 mm in diameter, were carefully incised using a razor blade. Subsequently, granulosa cells were delicately extracted from the inner surface of the cleansed follicles using a lancet. These cells were then isolated through centrifugation at 1500 rpm for 10 min. Following isolation, the granulosa cells were resuspended and maintained in sterile DMEM/F12 (1:1) medium (BioWhittaker; Lonza, Verviers, Belgium), which was supplemented with 10% fetal calf serum (Bio-West Inc., Logan, UT, USA) and a 1% antibiotic–antimycotic solution (Sigma-Aldrich, St. Louis, MO, USA). Cell quantification was performed using an automated cell counter (Thermo Fisher Scientific Inc., Waltham, MA, USA), and cell concentration was adjusted to a standardized density of 106 cells per milliliter of medium. The resulting cell suspension was aliquoted into various culture vessels depending on the intended assay: 24-well culture plates (Nunc™, Roskilde, Denmark) containing 1 mL of suspension per well for trypan blue staining and ELISA analyses; 96-well culture plates (Brand^®^, Wertheim, Germany) containing 100 μL per well for BrdU incorporation and cell death detection assays; or 16-well chamber slides (Nunc, Inc., International, Naperville, IL, USA) containing 100 μL per well for immunocytochemical evaluation. The cells were incubated at 37.5 °C in a 5% CO_2_ atmosphere until they achieved a 75% confluent monolayer, a process that typically spanned 2 to 3 days.

### 2.4. Preparation, Culture, and Processing of Follicular Fragments

In the second series of experiments, intended for the analysis of hormones by ovarian follicular fragments, the tissue near the ovary was eliminated, and the ovaries were opened with scissors at the site of entry of blood vessels. The internal connective tissue within the ovary was then delicately disrupted with a lancet to expose the follicles. These follicles were mechanically compressed and isolated from the adjacent connective tissues. Following isolation, the follicular walls were meticulously sectioned into thin, longitudinal fragments, each measuring 2–4 mm in diameter. Afterward, these fragments were washed thrice in sterile DMEM/F12 1:1 medium (BioWhittaker). For culturing, the fragments were placed in the same medium, supplemented with 10% fetal calf serum (FCS; Bio-West) and 1% antibiotic–antimycotic solution (Sigma-Aldrich), and incubated in 24-well culture plates (Nunc) at a volume of 1 mL per fragment per well. The follicular fragments were maintained in a controlled environment with 5% CO_2_ at 37.5 °C for 2 days.

### 2.5. Cell Viability Test

To evaluate granulosa cell viability, we employed the trypan blue exclusion assay (0.4%; Sigma-Aldrich, St. Louis, MO, USA), a widely validated method adapted from Perry et al. (1997) and Uzuner et al. (2018) [20,21]. Following incubation, the culture medium was aspirated from the plates, exposing the adherent granulosa cell monolayer. Cells were stained with trypan blue for 15 min, enabling the dye to permeate nonviable cells with compromised membranes. To preserve cellular morphology, the monolayer was fixed in 4% paraformaldehyde for 30 min, followed by three washes with physiological saline to remove the excess dye. Viability was assessed under a light microscope at 400× magnification, where nonviable (stained blue) cells were distinguished from viable (unstained) cells. The proportion of dead cells relative to the total cell population was calculated.

### 2.6. BrdU Assay

Cell proliferation was evaluated by measuring the incorporation of 5-bromo-2′-deoxyuridine (BrdU) into DNA during synthesis. This was performed using a colorimetric cell proliferation ELISA kit (Roche Diagnostics GmbH, Roche Applied Science, Mannheim, Germany) following the manufacturer’s protocol. The resulting reaction products were quantified by determining absorbance at 450 nm with an ELISA reader (Thermo Fisher Scientific Multiskan FC, Vantaa, Finland).

### 2.7. Analysis of Nuclear Apoptosis

Apoptotic activity in cells was assessed using the Cell Death Detection Kit (Roche Diagnostics, Indianapolis, IN, USA), according to the manufacturer’s instructions. This assay quantifies cytoplasmic DNA–histone fragments as a marker of apoptosis-induced cell death. The reaction products were measured by absorbance at 450 nm using an ELISA reader (Thermo Fisher Scientific).

### 2.8. Analysis of Proliferation and Cytoplasmic Apoptosis Markers

To investigate cellular dynamics within ovarian granulosa cells, we employed immunocytochemistry to detect key markers of proliferation and apoptosis, as adapted from established protocols [22]. Proliferation was assessed via proliferating cell nuclear antigen (PCNA), a hallmark of the S-phase of the cell cycle [23], and cyclin B1, which regulates the G2/M transition and mitotic progression [24,25]. Apoptosis was evaluated using bax and caspase 3, critical mediators of mitochondrial and cytoplasmic programmed cell death [26]. These markers provide insights into the balance between cell growth and attrition, pivotal in ovarian function. Cells were incubated with primary mouse monoclonal antibodies targeting PCNA, cyclin B1, bax, or caspase 3 (Santa Cruz Biotechnology, Inc., CA, USA; diluted 1:500 in PBS). A secondary swine anti-mouse IgG antibody (Santa Cruz Biotechnology, Inc.; diluted 1:1000), conjugated to horseradish peroxidase (HRP; Servac, Prague, Czech Republic), was then applied. HRP-labeled cells were visualized using 3,3′-diaminobenzidine (DAB) substrate (Roche Diagnostics GmbH, Mannheim, Germany), yielding a distinctive brown stain. Negative controls, processed without primary antibodies, confirmed staining specificity. Using light microscopy, we quantified the proportion of DAB-positive cells relative to the total cell population and noted the intracellular localization of each marker.

### 2.9. Immunoassay of Hormones

Progesterone and 17β-estradiol, pivotal regulators of ovarian cell proliferation, apoptosis, and folliculogenesis, oogenesis, and luteogenesis [27,28,29], were quantified to explore their roles in the experimental context. Aliquots (25 μL) of culture medium were analyzed using enzyme-linked immunosorbent assays (ELISA) with kits from LDN Immunoassays and Services (Nordhorn, Germany), following the manufacturer’s instructions. Assay performance metrics (sensitivity, specificity, and range) are detailed in Table 1. To ensure reliability in the culture medium matrix, we validated the ELISA using dilution linearity tests. This step confirmed the assays’ accuracy in detecting hormone concentrations, providing a robust measure of endocrine activity reflective of ovarian cellular responses under varying dietary conditions.

### 2.10. Sample Preparation for Proteomic Evaluation (Profiling)

For the nano HPLC-Chip-MS/MS analysis, cells from each experimental group were enzymatically dissociated using Accutase (Sigma-Aldrich) and subsequently washed twice with phosphate-buffered saline (PBS) to remove residual detachment agents and culture medium. The resultant cell suspension, adjusted to a concentration of 10^6^ cells/mL, was lysed in 200 µL of urea lysis buffer comprising 8 M urea, 0.1 M dithiothreitol (DTT), and a 1× concentration of protease inhibitors (Roche Complete). Lysis was facilitated by agitation for 1 h at 37 °C. Following lysis, the samples were immediately cooled to 4 °C and subjected to centrifugation at 16,000× *g* for 5 min to pellet cellular debris. The supernatant was carefully transferred to 3 kDa molecular weight cut-off filter plates (Amicon Ultra-0.5 mL 3K, Millipore, Burlington, MA, USA) and centrifuged at 14,000× *g* for 40 min to concentrate the protein content. To extract tryptic peptides from the complex protein mixture, a modified filter-aided sample preparation (FASP) protocol was employed, as described in reference [30]. Detergent removal and buffer exchange were achieved through two sequential washes of the filter plates, each using 200 µL of 8 M urea in 0.1 M Tris/HCl (pH 8.5, denoted as UA), followed by centrifugation at 14,000× *g* for 40 min per wash. The flow-through was discarded from the collection tubes after each step. Protein alkylation was subsequently performed by adding 100 µL of 0.05 M iodoacetamide in UA, with mixing at 600 rpm in a thermomixer for 1 min, followed by a 20 min incubation without agitation. The samples were then centrifuged at 14,000× *g* for 30 min to remove excess reagents. To eliminate residual urea and prepare the proteins for enzymatic digestion, two additional washes were conducted using 100 µL of 0.05 M ammonium bicarbonate (NH_4_HCO_3_), with centrifugation at 14,000× *g* for 10 min after each wash. Protein digestion was initiated by the addition of 2 µg of trypsin (Trypsin Gold, Promega, Cyprus, Greece) in 0.05 M NH_4_HCO_3_, followed by overnight incubation at 37 °C to ensure complete hydrolysis. The resulting peptides were recovered by inverting the filter plates and centrifuging at 14,000× *g* for 40 min. The peptide mixture was then vacuum-dried to remove the solvent, and the dried pellet was reconstituted in 50 µL of mobile phase consisting of 97% water and 3% acetonitrile, preparing it for subsequent nano HPLC-Chip-MS/MS analysis.

### 2.11. Protein Identification by Tandem Mass Spectrometry (Nano HPLC-Chip-MS/MS)

The tryptic peptides were introduced into a 40 nL enrichment column, packed with Zorbax SB C18 (5 μm particle size), integrated within the Agilent 1260 ChipCube MS Interface. This loading process was facilitated by an Agilent 1260 Capillary Pump (Agilent Technologies, Palo Alto, CA, USA). Following sample loading and subsequent desalting on the enrichment column, the peptides were eluted in forward-flush mode and transferred to the analytical column at a flow rate of 600 nL/min using an Agilent 1260 Nano Pump. The elution was driven by a gradient of increasing organic solvent concentration. The mobile phase comprised two components: an aqueous solution (A) and an acetonitrile-based solution (B), both containing 0.1% (*v*/*v*) formic acid. Chromatographic separation was accomplished through a gradient elution protocol programmed as follows: 0 min, 3% B; 2 min, 3% B; 25 min, 50% B; 30 min, 50% B; 35 min, 95% B; 40 min, 95% B; 45 min, 3% B, followed by a 10 min post-run period to allow for column re-equilibration. The analytical column was coupled with a Q-TOF Agilent 6500 Series mass spectrometer. A voltage of 1850 V was applied to the electrodes within the nanospray ionization chamber. High-purity nitrogen (99.99999%) served as the collision gas, with the collision energy dynamically adjusted based on the mass and charge of the precursor ions. Tandem mass spectrometry (MS/MS) spectra were collected through automated switching between MS and MS/MS modes (auto MS/MS mode). The resulting MS/MS data were processed using the SpectrumMill search engine (Agilent Technologies, Palo Alto, CA, USA). Database searches were conducted against a custom-built database derived from the UniProt rabbit (Oryctolagus cuniculus, Thorbecke inbred) proteome (UP000001811). Protein and peptide identifications were validated using the auto-validation criteria of the SpectrumMill software version B.04.01.141 which included a minimum protein score of 10. For spectra derived from the fragmentation of precursor ions with charge states of 2+, 3+, and 4+, the minimum scores were set at 8, 7, and 9, respectively, with a requirement that the scored peak intensity reach at least 60%.

### 2.12. Gene Ontology Enrichment Analysis

A compilation of UniProt IDs corresponding to proteins within each derived cluster served as the input dataset for a gene ontology (GO) enrichment analysis, encompassing the categories of biological process, cellular component, and molecular function. For this purpose, we employed ShinyGO version 0.82, an open-source, web-based tool accessible at https://bioinformatics.sdstate.edu/go/ (accessed on 1 December 2024) [31], designed for advanced functional enrichment analysis. This tool generates a variety of outputs, including hierarchical clustering trees, networks that consolidate overlapping terms and pathways, protein–protein interaction networks, plots depicting gene characteristics, and enriched promoter motifs. The extent of functional enrichment, expressed as fold changes, alongside the number of associated genes and their corresponding false discovery rate (FDR) values, is visually represented. A *p*-value threshold of 0.05 was established as the criterion for statistical significance.

### 2.13. Statistical Analysis

A statistical model of the weight of females from the NR and RF groups included the fixed effect of the moment (initial and final) and female, and error was included as a random effect. Statistical models of initial and final weights of the female, weight of the left ovary, weight of the right ovary, length and weight of the left uterine horn, and length and weight of the right uterine horn included a group effect (NF and RF) and the error.

From each ovary, at least 50 histological sections were prepared and analyzed. In the in vitro experiments, each experimental group was represented by four culture wells containing ovarian granulosa cells isolated from eight ovaries. Cell viability was assessed using the trypan blue exclusion test, with viability rates calculated based on a minimum of 100 cells per well. For immunocytochemical analysis, the proportion of antigen-positive cells was determined by evaluating at least 1000 cells per well. In the enzyme-linked immunosorbent assay (ELISA), blank control values were subtracted from the corresponding values obtained from media-containing cells to eliminate nonspecific background signals, which constituted less than 10% of the total values. The secretion rates of substances were expressed as the amount secreted per 105 viable cells per day. Additional analyses were conducted using eight samples per group. To determine statistically significant differences between groups, data were subjected to the Shapiro–Wilk test for normality and analyzed using Student’s *t*-test, one-way analysis of variance (ANOVA) with a repeated-measures design, where applicable, followed by Tukey’s post hoc test, or the Chi-square test. These analyses were performed using SigmaPlot 11.0 software (Systat Software GmbH, Erkrath, Germany). Differences were considered statistically significant at a probability level of *p* < 0.05.

## 3. Results

### 3.1. Fecundity and Anatomical Reproductive Traits of Does Subjected or Not Subjected to Food Restriction

Females showed similar weights at the beginning and end of the experimental period in both the NF and RF groups (Table 2). There was no significant difference between NF and RF female weights at the beginning of the experiment. At the end of the experiment, the weights of NF females were higher than those of RF females (Table 3). Weights of the ovaries were higher in the NF group than in the RF group for both the left and the right ovaries (31% and 33%, respectively). While the lengths of the right and the left uterine horns were longer in the RF group than in the NF group (around 21%), the weights of both uterine horns were lower (around 40%).

### 3.2. Histomorphometric Traits in Ovaries of Does Subjected to Normal or Restricted Feeding

About 45% of the follicles were dormant primordial follicles in both NF and RF groups (Table 4). No significant differences were found in follicle diameter, oocyte diameter, and percentage of degenerated follicles in dormant and growing primordial follicles. However, the RF group showed a higher proportion of dormant primary follicles and preovulatory follicles and a greater follicle diameter and thickness of the theca in secondary and preovulatory follicles than the NF group. The granulosa thickness of primary, secondary, and preovulatory follicles was higher in the RF group than in the NF group. Also, oocyte diameter was higher in the RF group than in the NF group for primary and secondary follicles. Percentages of degenerated follicles were similar between groups, except for primary follicles, whose proportion was higher in the NF group.

### 3.3. Viability, Proliferation, Apoptosis, and Release of Hormones by Ovarian Cells and Fragments of Does Subjected to Nonrestricted or Restricted Feeding

**Figure 2:**
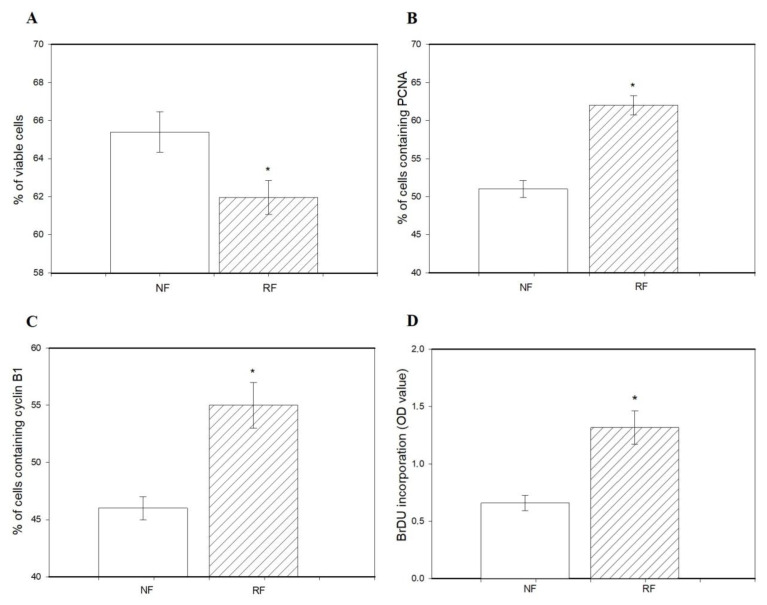
The percentage of viable cells (**A**), accumulation of PCNA (**B**), cyclin B1 (**C**), and BrDU (**D**) in ovarian granulosa cells isolated from the ovaries of rabbits subjected (RF) or not subjected to 50% caloric restriction (NF). Values represent means ± SEM. * indicates statistically significant differences (*p* < 0.05).

**Figure 3:**
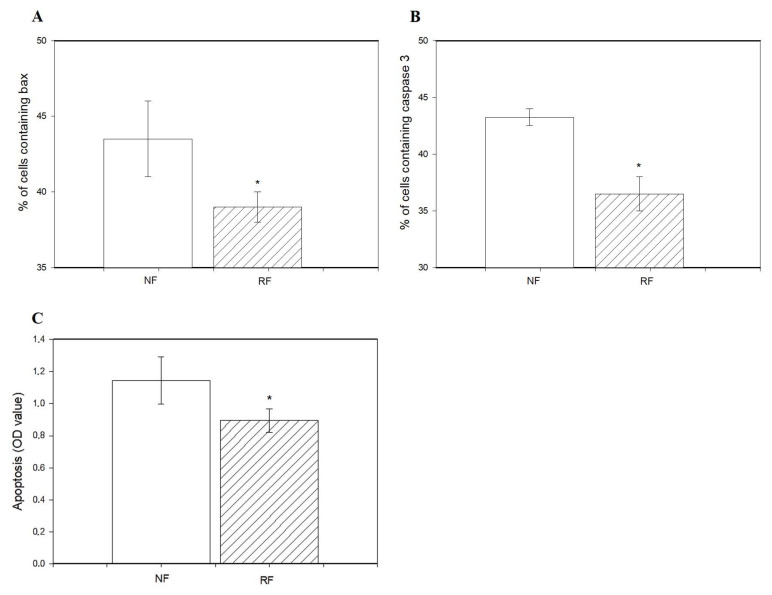
Accumulation of bax (**A**), caspase 3 (**B**), and index of apoptotic cell death in ovarian granulosa cells (**C**) from rabbits subjected (RF) or not subjected to 50% caloric restriction (NF). Values represent means ± SEM. * indicates statistically significant differences (*p* ≤ 0.05).

The percentage of viable cells was lower in granulosa cells isolated from the ovaries of the RF group than in the NF group (Figure 2A). Restricted feeding promoted the accumulation of PCNA (Figure 2B), cyclin B1 (Figure 2C), and BrDU (Figure 2D) and reduced accumulation of bax and caspase 3 (Figure 3A,B), as well as the occurrence of DNA-fragmented cells (Figure 3C) in ovarian cells.

**Figure 4:**
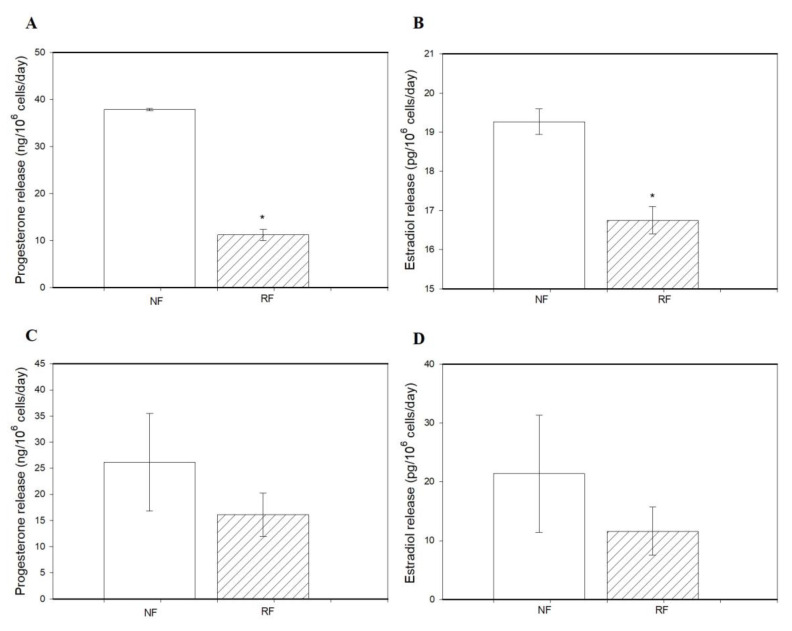
The release of progesterone and estradiol by ovarian granulosa cells (**A**,**B**) and ovarian fragments (**C**,**D**) of rabbits subjected (RF) or not subjected to 50% caloric restriction (NF). Values represent means ± SEM. * indicates statistically significant differences (*p* < 0.05).

Granulosa cells of the RF group released less P4 and E2 than those from the NR group (Figure 4A,B). However, the release of P4 and E2 by ovarian fragments was not affected by food restriction (Figure 4C,D).

### 3.4. Proteomic Analysis of Ovarian Cells of Does Subjected to Normal or Restricted Feeding

**Figure 5:**
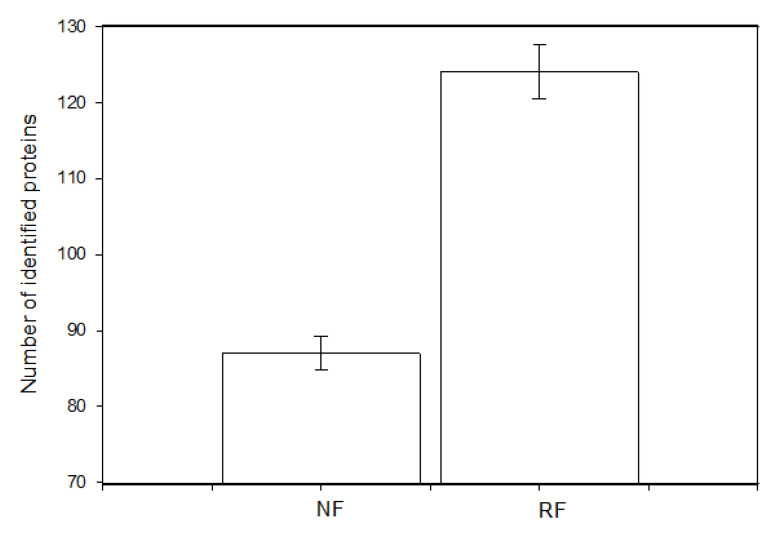
Differences in the number of identified proteins specific to granulosa cells isolated from the ovaries of does subjected to normal (NF) or restricted feeding (RF).

We were able to discern differences between the proteomic profiles of granulosa cells isolated from the ovaries of does in the NR and RF groups. The total number of identified proteins was 847, with the number of specific proteins for the NR group being 87 and for the RF group being 124 (Figure 5). The selected proteins that appeared to be differentially expressed in the granulosa cells of the two compared groups are listed in Table 5. Cells from the RF group produced more peptides involved in cell differentiation, cell proliferation/division (cytoskeletal proteins), mitotic cell cycle, antioxidant activity, carbohydrate metabolic processes, and protease inhibition than the NR group, although they exhibited reduced (i.e., below the detection level) accumulation of proteins involved in transporter activity, extracellular matrix organization, and programmed cell death (Table 5).

### 3.5. Functional Enrichment Analysis

**Figure 6:**
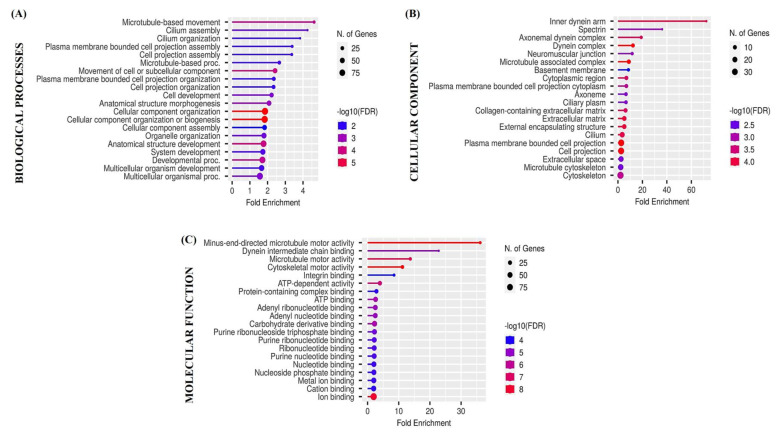
Lollipop plot of Gene Ontology (GO) enrichment analysis (biological processes (**A**), cellular components (**B**), and molecular functions (**C**)) results obtained using the ShinyGO v0.82 application. The points in each dot plot are sized according to the proportion of all proteins within the cluster annotated with the corresponding term and colored by enrichment confidence (FDR).

Biological processes, cellular components, and molecular functions of differentially expressed genes were detected using the ShinyGO version 0.82. The 20 enriched terms (*p* < 0.05) in each category are presented in Figure 6.

## 4. Discussion

### 4.1. Anatomical Characterisation of the Reproductive Tract of Females Under Food Restriction

The weights of females were slightly lower than those found in the literature, around 4.0 kg, possibly due to the different genetic origin of the females [32,33]. However, ovarian weights were similar [32]. Moderate and relatively short food restriction in our experiment did not affect body weight, as previously reported by Sirotkin et al. (2017) [14]. However, the final weights of the females and the ovarian weights were lower in feed-restricted females. When the weight of the ovary is expressed relative to the weight of the female, the ovary represents 0.22‰ of the doe weight in the unrestricted female group and 0.16‰ in the restricted female group, both values being higher than those found by Rebollar et al. (2009) [33] for females fed ad libitum and with a different genetic background (0.14‰).

The weight and length of the uterus were affected by food restriction. Total uterine length was 13.12 cm in females not subjected to food restriction. This result agrees with the length reported by [34]. However, food restriction increased the length of the uterus to 16.64 cm. This observation suggests that food restriction can reduce the thickness of the uterine horns while increasing their length. Argente et al. [35] analyzed the characteristics and irrigation of the uterine horns in two genetic lines with different uterine capacities. Their findings suggest that each embryo requires a minimum amount of space in the uterus to implant, survive, and develop. Additionally, they observed that the initial length and available uterine space particularly influence the development of the maternal placenta. Thus, it could be expected that food restriction would increase prenatal survival by increasing the length of the uterus. However, further histological studies of the uterine horn tissues would be necessary to corroborate this hypothesis and rule out dystrophic atrophy of the reproductive organs.

### 4.2. Effect of Food Restriction on Folliculogenesis and Oogenesis in the Ovaries

The size of both oocytes and follicles at all stages of oogenesis and folliculogenesis, respectively, is similar to that found by [36]. As expected, the sizes of the oocytes increased progressively from preantral follicles to preovulatory follicles [37]. The oocyte maturation rate depends on its initial size. Small oocytes are unable to mature; however, those with a diameter exceeding 80% of their final size have the capacity to resume the meiotic process [38]. In our experiment, oocyte diameter in the primary follicles was 74% of their size in the preovulatory follicles in the NF group, while it was 87% in RF females. Thus, the oocytes collected from the restricted group will be able to resume meiotic activity.

We found a high and similar number of dormant and growing primordial follicles in both feeding groups. Therefore, the fifth delivery in rabbits was far from being the endpoint of the female’s reproductive lifespan. The lack of differences between groups in the diameter of primordial follicles and oocytes suggests that, at this stage, no differentiation in their growth occurs.

The transition from primordial to early primary follicle coincides with a change in granulosa cell morphology and a significant increase in oocyte diameter in restricted females. During activation, the enlargement of both the oocyte and granulosa cells occurs simultaneously, ultimately leading to the formation of the early primary follicle. This suggests that early primary follicles are indeed part of the growing follicle pool [39]. The larger diameter of oocytes and increased thickness of granulosa cells in the RF group from preantral follicles to preovulatory follicles indicates the more intensive growth of oocytes in this group. Granulosa cells supply nutrients and metabolites to oocytes through cleavage junctions and secrete paracrine signals to regulate oocytes [40]; therefore, the increased growth of oocytes could indicate more cytoplasmatic maturation [27]. Therefore, increased granulosa thickness in food-restricted females would improve oocyte quality. Our results demonstrate that food restriction promotes the growth of primary and secondary follicles, as well as their selection and development into preovulatory follicles.

### 4.3. Effect of Food Restriction on Proliferation, Apoptosis, and Hormone Release by Ovarian Cells

In the present study, food restriction inhibited cell viability and apoptosis (accumulation of bax and caspase 3, and DNA fragmentation) and promoted proliferation (accumulation of PCNA and cyclin B1, and BrdU incorporation) of rabbit ovarian cells.

It is well-known that BrdU is an exogenous marker of DNA synthesis [41], PCNA is an endogenous marker/promoter of the S-phase of the cell cycle, and cyclin B1 is an endogenous marker/promoter of the G2-M phase mitosis [22,23]. Therefore, we suggest that food restriction may affect both types of cell proliferation. Moreover, the results from our study also showed that food restriction reduced the percentage of DNA-fragmented cells and accumulation of bax and caspase 3 (markers of cytoplasmic apoptosis [24]). Therefore, these observations indicate that restricted-feeding diets may increase not only a synthesis of DNA and reduction of DNA fragmentation, but also the transition between particular phases of mitosis.

It is well known that nutrition affects intracellular regulators of ovarian cell functions via metabolic or reproductive hormones [4]. In our experiment, food restriction reduced the output of P4 and E2 by ovarian cells. The present findings are consistent with a previous report by McCann and Hansel [42], who demonstrated the ability of restricted feeding to decrease progesterone in heifers. On the other hand, when examining the capacity of ovarian fragments to secrete steroid hormones, no significant differences were observed between the groups.

Further elucidation is necessary to understand the functional interrelationships among various ovarian processes in the studied animals. Nonetheless, the current observations suggest that variations in ovarian folliculogenesis, steroidogenesis, cellular proliferation, and apoptosis may account for the differences observed between the studied groups.

### 4.4. Effect of Food Restriction on Proteomic Profile

Our study found that the RF group produced more specific proteins than its NF counterpart (124 vs. 87), including one peptide involved in cell differentiation, eight involved in cell proliferation/division (cytoskeletal proteins), one involved in the mitotic cell cycle, two involved in GTP-ase activity, and one involved in antioxidant activity, regulation of DNA-templated transcription, carbohydrate processes, and protease inhibition, but without detection for extracellular matrix organization and programmed cell death. Such proteins are important regulators of oocyte maturation, early mammalian embryo development, and maternal immunity [43,44,45].

These observations suggest that food restriction in rabbits may affect multiple peptides that govern various biological processes. Furthermore, the absence of certain specific regulatory proteins in both normal-feeding and restricted-feeding groups suggests that fecundity may be governed not only by the presence but also by the absence or underproduction of certain proteins that either stimulate or inhibit various cellular functions. The current findings imply that fecundity in rabbits is subject to multiple regulatory mechanisms. However, the correlation between fecundity and the production of specific proteins offers only indirect evidence of their involvement in ovarian functions and fecundity. Additional research is necessary to elucidate the roles and functional interrelationships among the identified proteins and their targets.

In summary, these findings are novel because they demonstrate the influence of food restriction not only on the marker of the S-phase of the cell cycle (PCNA), but also on the marker of the G-phase of mitosis (cyclin B1) and on cell proliferation itself (BrdU incorporation). Additionally, they reveal the impact of food restriction not only on cytoplasmic/mitochondrial apoptosis, indicated by bax accumulation, but also on nuclear apoptosis through DNA fragmentation. Furthermore, for the first time, our findings establish the effect of food restriction on ovarian cell viability, which, in turn, influences ovarian follicular development, selection, and fecundity. They also provide the first piece of evidence of the impact of food restriction on ovarian folliculogenesis at different stages. Lastly, through proteomic analysis, they identify a large number of regulatory peptides and the corresponding pathways affected by food restriction.

Therefore, the present data substantially expand the available knowledge concerning mechanisms of food restriction effects on fecundity. Therefore, our results could be used to optimize the diets of female rabbits and determine feeding rates in order to simultaneously improve reproductive performance and reduce feed costs.

## 5. Conclusions

In conclusion, food restriction could activate rabbit female reproduction by (1) selection of the growing primordial ovarian follicles, (2) better transformation of secondary into preovulatory follicles, (3) increasing the growth (cytoplasmic maturation) of oocytes, (4) increasing proliferation and decreasing apoptosis in ovarian granulosa cells, (5) changes in ovarian secretory activity, and (6) changes in the number of peptides involved in cell differentiation, proliferation/division (cytoskeletal proteins), and adhesion. Further studies are necessary to confirm these changes, as the main criterion for assessing the effectiveness of the impact of food restriction should be the production of live offspring.

## Figures and Tables

**Table 1:** Characteristics of the immunoassays used in the experiments.

Substance Assayed	Specificity of Assay (Cross-Reactivity of Antiserum)	Sensitivity of Assay (ng/mL)	Coefficient of Variation (%)	
			Intra-Assay	Inter-Assay
Progesterone	≤1.1% with 11-desoxycorticosterone, ≤0.35% with pregnenolone, ≤0.30% 17α-OH with progesterone, ≤0.20% with corticosterone, ˂0.10% with estriol, 17β-estradiol, testosterone, cortisone, and 11-desoxycortisol, ˂0.02% with DHEA-S and cortisol	0.045	5.4	5.6
17β-estradiol	≤9.5% with fulvestrant, ≤4.2% with estrone, ≤3.8% with E2-3-glucuronide, ≤3.6% with E2-3-sulphate, ≤0.4% with estriol, ˂0.1% with androstenedione, 17-hydroxyprogesterone, corticosterone, pregnenolone, E2-17-glucuronide, progesterone, and testosterone	0.0062	6.4	4.5

**Table 2:** Initial and final weights of does subjected to food restriction (RF) or not subjected to food restriction (NF).

Traits		Initial (n = 8)	Final (n = 8)	*p*
Female weight	NF (g)	3532 ± 171	3766 ± 175	ns
	RF (g)	3349 ± 125	3190 ± 104	ns

The values are means ± SEM. Ns: no significant differences between initial and final weights.

**Table 3:** Anatomical reproductive traits of does subjected to food restriction (RF) or not subjected to food restriction (NF).

Traits		NF (n = 8)	RF (n =8)	*p*
Female weight	Initial (g)	3532 ± 171	3349 ± 125	ns
	Final (g)	3766 ± 175	3190 ± 104	**
Ovary weight	Left (g)	0.36 ± 0.05	0.25 ± 0.03	*
	Right (g)	0.40 ± 0.05	0.27 ± 0.03	*
Uterine horn length	Left (cm)	6.56 ± 0.30	8.40 ± 0.66	*
	Right (cm)	6.56 ± 0.43	8.24 ± 0.52	*
Uterine horn weight	Left (g)	5.09 ± 0.35	2.90 ± 0.37	***
	Right (g)	4.92 ± 0.44	3.08 ± 0.40	**

The values are means ± SEM. * *p* < 0.05; ** *p* < 0.01; *** *p* < 0.001 significant differences between groups (NF and RF).

**Table 4:** The results of histomorphometric analysis of indexes of folliculogenesis and oogenesis in ovaries of does subjected to nonrestricted (NF) or restricted feeding (RF).

Ovarian Structures and Their Parameters	Feeding	
	NF	RF
Dormant primordial follicles:		
Number	126	176
Proportion concerning all detected follicles (%)	45	48.2
Diameter of follicles (µm)	30.59 ± 0.45	32.01 ± 0.36 *
Diameter of oocytes (µm)	23.21 ± 0.36	24.54 ± 0.35
% of degenerated follicles	0	0
Growing primordial follicles:		
Number	64	70
Proportion concerning all detected follicles (%)	22.9	19.18
Diameter of follicles (µm)	55.62 ± 1.72	58.69 ± 1.89
Diameter of oocytes (µm)	35.44 ± 1.28	36.24 ± 1.27
% of degenerated follicles	18.8	20.0
Primary (preantral) follicles:		
Number	52	74
Proportion concerning all detected follicles (%)	18.57	20.27
Diameter of follicles (µm)	182.27 ± 7.54	170.72 ± 5.57
Thickness of theca (µm)	17.62 ± 0.54	16.51 ± 1.06
Thickness of granulosa (µm)	31.65 ± 1.06	34.56 ± 1.82 *
Diameter of oocytes (µm)	84.90 ± 2.70	103.14 ± 4.91 *
% of degenerated follicles	7.7	25.7 *
**Secondary (antral) follicles:**		
Number	36	39
Proportion concerning all detected follicles (%)	12.86	10.68
Diameter of follicles (µm)	403.95 ± 15.36	460.75 ± 31.09 *
Thickness of theca (µm)	41.76 ± 1.56	45.61 ± 1.11 *
Thickness of granulosa (µm)	110.80 ± 1.71	127.65 ± 0.84 *
Diameter of oocytes (µm)	103.02 ± 3.81	114.25 ± 2.49 *
% of degenerated follicles	27.8	30.8
**Preovulatory follicles:**		
Number	2	6
Proportion concerning all detected follicles (%)	0.71	1.64 *
Diameter of follicles (µm)	429.53 ± 63.47	824.78 ± 101.56 *
Thickness of theca (µm)	33.27 ± 1.60	58.15 ± 3.10 *
Thickness of granulosa (µm)	124.52 ± 6.12	294.79 ± 35.94 *
Diameter of oocytes (µm)	113.93 ± 11.12	118.89 ± 3.26
% of degenerated follicles	0	0

The values are means ± SEM. * indicates statistically significant (*p* < 0.05) differences between groups (nonrestricted and restricted feeding).

**Table 5:** The selected proteins differently expressed in granulosa cells of does subjected (RF) or not subjected (NF) to food restriction and the function of these proteins.

Protein Function	Accession Number *	Protein Name	Feeding	
			NF	RF
Cell differentiation	G1SDQ4	Rho guanine nucleotide exchange factor 28	-	+
Cell growth	G1SD01 G1TV13 A0A5F9CH25 G1T8R3 U3KPK3 A0A5F9C3V6 A0A5F9CT68 G1SU35 A0A5F9CHT0 B7NZQ3 A0A5F9C3J9	Spectrin beta chain Dynein axonemal heavy chain 2 Dynein axonemal heavy chain 17 Utrophin Diaphanous related formin 2 Myosin XVB Cytoskeleton associated protein 5 Dynein axonemal heavy chain 1 Ubiquitinyl hydrolase 1 Deoxyribonuclease Cell migration inducing hyaluronidase 1	- - - - - + + + - - -	+ + + + + - - -+ + + +
Transporter activity	G1SLC9	Sodium/hydrogen exchanger	+	-
Extracellular matrix organization	A0A5F9C8H0	ADAM metallopeptidase with thrombospondin type 1 motif 9	+	-
Programmed cell death	Q27Q52	Protein Wnt-5a	+	-
Mitotic cell cycle	A0A5F9C8V0	Tetratricopeptide repeat domain 28	-	+
GTP-ase activity	A0A5F9CV79 G1STZ5 G1U5G0	Neurofibromin 1 Nonspecific serine/threonine protein kinase Rho guanine nucleotide exchange factor 33	- - +	+ + -
Antioxidant activity	A0A5F9DGD9	Peroxidasin like	-	+
Regulation of DNA-templated transcription	A0A5F9CNT4 A0A5F9C9Q6	GLI family zinc finger 3 Zinc finger protein 7	- +	+ -
Carbohydrate metabolic processes	A0A5F9CRS6	Otogelin like	-	+
Protease inhibition	A0A5F9C8I9	Alpha-2-macroglobulin	-	+
Chromatin organization	A0A5F9DIU5	Jumonji domain containing 1C	+	-

* Protein accession number refers to the UniProt database. +: detected, -: undetected (below the detection limit).

## Data Availability

All data are available upon request.

## Abbreviations

The following abbreviations are used in this manuscript:

NF	Females fed ad libitum
RF	Females subjected to 50% food restriction
DAB	3.3′-diaminobenzidine substrate

## References

[1] Boland M.P., Lonergan P., O’Callaghan D. (2001). Effect of nutrition on endocrine parameters, ovarian physiology and oocyte and embryo development. Theriogenology.

[2] Ferguson E.M., Ashworth C.J., Edwards S.A., Hawkins N., Hepburn N., Hunter M.G. (2003). Effect of different nutritional regimens before ovulation on plasma concentrations of metabolic and reproductive hormones and oocyte maturation in gilts. Reproduction.

[3] Kiyma Z., Alexander B.M., Van Krik E.A., Murdoch W.J., Hallford D.M., Moss G.E. (2004). Effects of feed restriction on reproductive and metabolic hormones in ewes. J. Anim. Sci..

[4] Brecchia G., Bonanno A., Galeati G., Federici C., Maranesi M., Gobbetti A., Boiti C. (2006). Hormonal and metabolic adaptation to fasting: Effects on the hypothalamic-pituitary-ovarian axis and reproductive performance of rabbit does. Domest. Anim. Endocrinol..

[5] Armstrong D.G., McEvoy T.G., Baxter G., Robinson J.J., Hogg C.O., Woad K.J., Webb R., Sinclair K.D. (2001). Effect of dietary energy and protein on bovine follicular dynamics and embryo production in vitro: Associations with the ovarian insulin-like growth factor system. Biol. Reprod..

[6] Alexander B.M., Kiyma Z., McFarland M., Van Krik E.A., Hallford D.M., Hawkins D.E., Kane K.K., Moss G.E. (2007). Influence of short-term fasting during the luteal phase of the oestrous cycle on ovarian follicular development during the ensuing proestrus of ewes. Anim. Reprod. Sci..

[7] Szendrő Z., Szendrő K., Zotte A.D. (2012). Management of reproduction on small, medium and large rabbit farms: A review. Asian Australas. J. Anim. Sci..

[8] Manal A.F., Tony M.A., Ezzo O.H. (2010). Feed restriction of pregnant nulliparous rabbit does: Consequences on reproductive performance and maternal behaviour. Anim. Reprod. Sci..

[9] García-García R.M., Rebollar P.G., Arias-Alvarez M., Sakr O.G., Bermejo-Alvarez P., Brecchia G., Gutierres-Adan A., Zerani M., Boiti C., Lorenzo P.L. (2011). Acute fasting before conception affects metabolic and endocrine status without impacting follicle and oocyte development and embryo gene expression in the rabbit. Reprod. Fertil. Dev..

[10] Menchetti L., Brecchia G., Canali C., Cardinali R., Polisca A., Zerani M., Boiti C. (2015). Food restriction during pregnancy in rabbits: Effects on hormones and metabolites involved in energy homeostasis and metabolic programming. Res. Vet. Sci..

[11] Fortun-Lamothe L. (1998). Effects of pre-mating energy intake on reproductive performance of rabbit does. Anim. Sci..

[12] Daoud N.M., Mahrous K.F., Ezzo O.H. (2012). Feed restriction as a biostimulant of the production of oocyte, their quality and GDF-9 gene expression in rabbit oocytes. Anim. Reprod. Sci..

[13] Naturil-Alfonso C., Lavara R., Vicente J.S., Marco-Jimenez F. (2016). Effects of female dietary restriction in a rabbit growth line during rearing on reproductive performance and embryo quality. Reprod. Domest. Anim..

[14] Sirotkin A.V., Koničková I., Østrup O., Rafay J., Laurincik J., Harrath A.H. (2017). Caloric restriction and IGF-I administration promote rabbit fecundity: Possible interrelationships and mechanisms of action. Theriogenology.

[15] Harrath A.H., Østrup O., Rafay J., Koničková Florkovičová I., Laurincik J., Sirotkin A.V. (2017). Metabolic state defines the response of rabbit ovarian cells to leptin. Repro. Biol..

[16] Blasco A., Martínez-Álvaro M., García M.L., Ibáñez-Escriche N., Argente M.J. (2017). Selection for genetic environmental sensitivity of litter size in rabbits. Genet. Sel. Evol..

[17] Pedersen T., Peters H. (1968). Proposal for a classification of oocytes and follicles in the mouse ovary. J. Reprod. Fertil..

[18] Sirotkin A.V., Pavlova S., Tena-Sempere M., Grossmann R., Jiménez M.R., Rodriguez J.M., Valenzuela F. (2013). Food restriction, ghrelin, its antagonist and obestatin control expression of ghrelin and its receptor in chicken hypothalamus and ovary. Comp. Biochem. Physiol. A Mol. Integr. Physiol..

[19] Mescher A.L. (2021). Junqueira’s Basic Histology: Text and Atlas.

[20] Perry S.W., Epstein L.G., Gelbard H.A. (1997). In situ trypan blue staining of monolayer cell cultures for permanent fixation and mounting. BioTechniques.

[21] Uzuner S.Ç. (2018). Development of a direct trypan blue exclusion method to detect cell viability of adherent cells into ELISA plates. Celal Bayar Univ. Fen Bilim. Derg..

[22] Fabová Z., Loncová B., Mlynček M., Sirotkin A.V. (2022). Interrelationships between amphiregulin, kisspeptin, FSH and FSH receptor in promotion of human ovarian cell functions. Reprod. Fertil. Dev..

[23] Moldovan G.L., Pfander B., Jentsch S. (2007). PCNA, the maestro of the replication fork. Cell.

[24] Dai Y., Jin F., Wu W., Kumar S.K. (2019). Cell cycle regulation and hematologic malignancies. Blood Sci..

[25] Ligasová A., Frydrych I., Koberna K. (2023). Basic Methods of Cell Cycle Analysis. Int. J. Mol. Sci..

[26] Spitz A.Z., Gavathiotis E. (2022). Physiological and pharmacological modulation of BAX. Trends Pharmacol. Sci..

[27] Sirotkin A.V. (2014). Regulators of Ovarian Functions.

[28] Chou C.H., Chen M.J. (2018). The effect of steroid hormones on ovarian follicle development. Vitam. Horm..

[29] Chen P., Li B., Ou-Yang L. (2022). Role of estrogen receptors in health and disease. Front. Endocrinol..

[30] Wiśniewski J.R., Zougman A., Nagaraj N., Mann M. (2009). Universal sample preparation method for proteome analysis. Nat. Methods.

[31] Ge S.X., Jung D., Yao R. (2020). ShinyGO: A Graphical Gene-Set Enrichment Tool for Animals and Plants. Bioinformatics.

[32] García M.L., Muelas R., Argente M.J., Peiró R. (2021). Relationship between prenatal characteristics and body condition and endocrine profile in Rabbits. Animals.

[33] Rebollar P.G., Pérez-Cabal M.A., Pereda N., Lorenzo P.L., Arias-Álvarez M., García-Rebollar P. (2009). Effects of parity order and reproductive management on the efficiency of rabbit productive systems. Livest. Scie..

[34] Bolet G., Garreau H., Joly T., Theau-Clement M., Falieres J., Hurtaud J., Bodin L. (2007). Genetic homogenisation of birth weight in rabbits: Indirect selection response for uterine horn characteristics. Livest. Scie..

[35] Argente M.J., Santacreu M.A., Climent A., Blasco A. (2006). Influence of available uterine space per fetus on fetal development and prenatal survival in rabbits selected for uterine capacity. Livest. Scie..

[36] Žitný J., Massányi P., Trakovická A., Rafaj J., Toman R. (2004). Quantification of the ovarian follicular growth in rabbits. Bull. Vet. Inst. Pulawy.

[37] Al-Mufti W., Bomsel-Helmreich O., Chritidès J.P. (1988). Oocyte size and intrafollicular position in polyovular follicles in rabbits. J. Reprod. Fert..

[38] Naseer Z., Ahmad E., Epikmen E.T., Uçan U., Boyacioğlu M., İpek E., Akosy M. (2017). Quercetin supplemented diet improves follicular development, oocyte quality, and reduces ovarian apoptosis in rabbits during summer heat stress. Theriogenology.

[39] Hutt K.J., McLaughlin E.A., Holland M.K. (2006). Primordial follicle activation and follicular development in the juvenile rabbit ovary. Cell Tissue Res..

[40] Alam M.H., Miyano T. (2020). Interaction between growing oocytes and granulosa cells in vitro. Reprod. Med. Biol..

[41] Martí-Clúa J. (2021). Incorporation of 5-Bromo-2′-deoxyuridine into DNA and Proliferative Behavior of Cerebellar Neuroblasts: All That Glitters Is Not Gold. Cells.

[42] McCann J.P., Hansel W. (1986). Relationship between insulin and glucose metabolism and pituitary-ovarian functions in fasted heifers. Biol. Reprod..

[43] Dunkley S., Scheffler K., Mogessie B. (2022). Cytoskeletal form and function in mammalian oocytes and zygotes. Curr. Opin. Cell Biol..

[44] Pathirana A., Diao M., Huang S., Zuo L., Liang Y. (2016). Alpha 2 macroglobulin is a maternally-derived immune factor in amphioxus embryos: New evidence for defense roles of maternal immune components in invertebrate chordate. Fish Shellfish Immunol..

[45] Saadeldin I.M., Tukur H.A., Aljumaah R.S., Sindi R.A. (2021). Rocking the boat: The decisive roles of rho kinases during oocyte, blastocyst, and stem cell development. Front. Cell Dev. Biol..

